# Multiplanar Skeletal and Dentoalveolar Changes Associated with Orthopedic and Surgically Assisted Maxillary Expansion: A Multicenter Retrospective Observational Study

**DOI:** 10.3390/bioengineering13101221

**Published:** 2026-10-19

**Authors:** Lucia Giannini, Marco Farronato, Gianna Dipalma, Angelo Michele Inchingolo, Cinzia Maspero

**Affiliations:** 1 Fondazione IRCCS Cà Granda Ospedale Maggiore Policlinico, 20122 Milan, Italycinzia.maspero@unimi.it (C.M.); 2 Department of Biomedical, Surgical and Dental Sciences, School of Dentistry, University of Milan, 20122 Milan, Italy; 3 Department of Interdisciplinary Medicine, University of Bari Aldo Moro, 70121 Bari, Italy; 4 Department of Life Science, Health and Health Professions, Link Campus University, 00165 Rome, Italy

**Keywords:** maxillary transverse deficiency, rapid palatal expansion, surgically assisted rapid palatal expansion, cephalometry, dentoalveolar changes

## Abstract

Background: This retrospective multicenter study describes skeletal and dentoalveolar changes following orthopedic rapid palatal expansion (RPE) in growing patients and surgically assisted expansion in skeletally mature adults. Objective: To characterize within-cohort transverse, sagittal, and vertical changes without directly comparing treatment effectiveness between clinically distinct populations. Methods: Records from two Italian centers (January 2024–December 2025) included 54 growing patients treated with orthopedic RPE and 27 nongrowing adults treated with surgically assisted expansion. Lateral and posteroanterior cephalograms and dental casts were assessed at baseline (T0), after appliance removal (T1), and, when available, 1 year later (T2). Changes were summarized within each cohort using mean differences and 95% confidence intervals. T2 cephalometric data were available for 20 orthopedic and 18 surgically assisted patients; dental-cast follow-up was available for 18 per cohort. Results: T0–T1 transverse increases predominated. In the orthopedic cohort, piriform aperture width increased by 1.72 mm (95% CI 1.32–2.12), maxillary skeletal width (MXR–MXL) by 3.56 mm (2.84–4.28), and cephalometric maxillary intermolar width by 6.56 mm (5.79–7.33). The corresponding surgically assisted changes were 1.17 mm (0.87–1.47), 1.39 mm (0.76–2.02), and 5.43 mm (4.95–5.91), respectively. During T1–T2, upper-intermolar cephalometric changes were +0.42 mm (95% CI −0.13 to 0.97) in the orthopedic cohort and −0.38 mm (−0.80 to 0.04) in the surgical cohort; dental-cast changes were +0.44 mm (−0.12 to 1.00) and −0.41 mm (−0.80 to −0.02), respectively. Follow-up summaries were exploratory and limited by incomplete ascertainment. Conclusions: Both clinically distinct cohorts showed maxillary transverse widening between T0 and T1. Changes from T1 to T2 were generally small in the available intermolar summaries, but incomplete follow-up precludes conclusions about treatment stability or relapse. Differences in skeletal maturity, treatment indication, and measurement landmarks, together with the retrospective design, do not permit direct causal or comparative-effectiveness inferences.

## 1. Introduction

Transverse maxillary deficiency is a common skeletal discrepancy that often presents as unilateral or bilateral posterior crossbite and may be dental, skeletal, or mixed in origin [1,2]. Treatment aims to restore transverse maxillomandibular relationships; previous studies have also investigated long-term expansion effects [3], dental-arch changes [4], and nasal-septum changes [5].

Rapid palatal expansion (RPE) applies orthopedic forces across the midpalatal suture. Potential effects on airway dimensions and sleep-disordered breathing have also been investigated [6]. Midpalatal sutural ossification and skeletal maturation influence the orthopedic response [7], while the use of maxillary expansion in obstructive sleep apnea has been reviewed separately [8]. Appliance design and activation protocols may modify dental and arch responses [9,10].

With increasing skeletal maturation, resistance of the midpalatal and circummaxillary sutures increases, and conventional orthopedic expansion may become less predictable at the skeletal level, with a larger dentoalveolar contribution. Classic observations describe skeletal and dental changes following midpalatal suture opening [11], while surgically assisted rapid palatal/maxillary expansion (SARPE/SARME) is an option for skeletally mature patients, with technique-dependent response and stability [12]. Reviews address the effects of expansion on circummaxillary structures [13], and surgical-orthodontic literature discusses transverse dental-arch stability [14] and presurgical orthodontic guidance [15].

Different outcomes have been investigated following expansion. Systematic and three-dimensional studies describe changes in nasal-cavity and upper-airway dimensions [16,17] and nasomaxillary complex volume in children with obstructive sleep apnea [18]. Additional studies document facial appearance changes [19], posteroanterior cephalometric transverse effects [20], and immediate skeletal and dental differences between expander designs [21]. The available evidence, therefore, concerns partly distinct anatomical outcomes, and should not be interpreted as demonstrating equivalent skeletal effects across all measurement sites.

The literature also addresses surgically assisted expansion within orthodontic-surgical treatment planning [22] and its sagittal and vertical effects [23]. Sagittal and vertical responses to transverse–sagittal maxillary expansion have been assessed across different malocclusion groups [24]. Early palatal expansion has also been investigated for correcting maxillary crowding compared with later orthodontic treatment [25]. Nasal-cavity and upper-airway outcomes have been examined separately in three-dimensional studies [26]. Treatment timing is particularly relevant: RPE performed before the pubertal growth peak has been associated with a greater skeletal contribution [27], while long-term reviews [28] and imaging studies [29,30] describe the distribution and persistence of skeletal and dentoalveolar changes. Reviews of surgically assisted expansion in adults describe heterogeneous techniques, skeletal and dental outcomes, and limitations of long-term stability evidence [31,32,33,34,35,36].

The principal knowledge gap is, therefore, not whether both approaches can widen the maxilla, but how skeletal and dentoalveolar changes are distributed across the transverse, sagittal, and vertical planes when clinically indicated expansion protocols are evaluated in populations at different stages of craniofacial development. Because growing patients and skeletally mature adults represent different clinical populations, placing their observations within the same study is useful for descriptive contextualization but does not constitute a comparative effectiveness design.

Accordingly, the research question was whether the pattern, magnitude, and precision of observed dentoskeletal changes differ across planes within each clinical cohort and what limited information is available regarding subsequent follow-up. The aim was to characterize these changes retrospectively in adults treated with surgically assisted expansion and, in parallel, in pediatric/adolescent patients treated with orthopedic RPE. No hypothesis of superiority, equivalence, or direct treatment-effect comparison between the cohorts was prespecified.

## 2. Materials and Methods

### 2.1. Study Population and Eligibility

This retrospective, nonrandomized, multicenter observational study included consecutive eligible clinical records of patients treated between January 2024 and December 2025 for transverse maxillary deficiency associated with posterior crossbite. Inclusion criteria were (1) a clinical diagnosis of transverse maxillary deficiency with posterior crossbite; (2) treatment with the protocol corresponding to skeletal growth status, namely surgically assisted rapid palatal expansion in skeletally mature/nongrowing adult patients or orthopedic rapid palatal expansion in pediatric/adolescent patients with remaining growth potential; and (3) availability of baseline (T0) and post-treatment (T1) clinical and radiographic documentation adequate for the planned cephalometric and/or dental-cast measurements. Subsequent follow-up (T2) was not required for inclusion. Patients were excluded when the transverse discrepancy/posterior crossbite was not documented, when an expansion protocol other than the two study protocols had been used, or when baseline and T1 records were insufficient for outcome assessment. No additional exclusion criteria were documented in the available source records. Skeletal maturity was assessed at diagnosis by cervical vertebral maturation (CVM) on the lateral cephalometric radiograph, which was available for every patient as part of the routine diagnostic records; when additional confirmation was clinically indicated, hand–wrist radiography was used as a complementary assessment. Thus, classification as “growing” or “nongrowing” was based on skeletal-maturity assessment, rather than chronological age alone.

The inclusion period comprised patients treated from January 2024 through December 2025. Orthodontic treatment histories were available for all included patients. None of the 54 patients in the orthopedic cohort had undergone previous orthodontic treatment. In the surgically assisted cohort, 9 of 27 patients (33.3%) had received previous orthodontic treatment, none of which involved maxillary expansion. Presurgical orthodontic decompensation, when performed as part of the current expansion protocol, was distinguished from previous orthodontic treatment, and is described below.

Group 1 comprised 27 adult patients classified as skeletally mature/nongrowing, on the basis of skeletal-maturity assessment (15 females and 12 males), aged 21–35 years, all presenting with posterior crossbite secondary to transverse maxillary deficiency, and treated with surgically assisted rapid palatal expansion. The existing dataset contained detailed sagittal and vertical skeletal-pattern classifications for 18 of these patients: 3 skeletal Class II patients with skeletal open bite; 6 skeletal Class I patients (3 normodivergent and 3 with skeletal open bite); and 9 skeletal Class III patients (3 with skeletal deep bite, 3 normodivergent, and 3 with skeletal open bite). These subgroup data were therefore interpreted descriptively, and were not extrapolated to the full surgical cohort.

Group 2 comprised 54 pediatric/adolescent patients classified as growing, on the basis of skeletal-maturity assessment (27 females and 27 males), aged 5–17 years, all presenting with posterior crossbite secondary to transverse maxillary deficiency, and treated with orthopedic rapid palatal expansion. This wide chronological age range spans different phases of craniofacial development. CVM assessment was available for all patients, and was supplemented by hand–wrist radiography when clinically indicated. The CVM-stage distribution in the orthopedic cohort was approximately 30% CS2, 50% CS3, and 20% CS4. Stage-specific outcome analyses were not prespecified, so the contribution of differences in maturation stage within the pediatric cohort could not be quantified separately from normal growth and treatment-associated change. Twenty-one patients had skeletal Class I (9 with skeletal deep bite, 3 normodivergent, and 9 with skeletal open bite), 15 had skeletal Class II (3 with skeletal deep bite and 12 with skeletal open bite), and 18 had skeletal Class III (9 with skeletal deep bite, 6 normodivergent, and 3 with skeletal open bite).

Skeletal-pattern classification was available for 18 of 27 surgically assisted patients; therefore, those counts are not extrapolated to the full surgical cohort. Age is reported as the available range because individual age-distribution summaries were not retained in the analytical dataset. Baseline transverse-deficiency severity, crossbite laterality/severity, and patient-level cumulative expansion amount were not systematically retained in the analytical dataset; these unavailable variables are identified explicitly, rather than reconstructed retrospectively (Table 1).

All participants, or their legal guardians when applicable, received detailed information about the study objectives and provided written informed consent prior to participation.

Participants were recruited among patients referred for orthodontic evaluation at the Fondazione IRCCS Ca’ Granda Ospedale Maggiore Policlinico, Milan, Italy, and at the Policlinico of Bari, Bari, Italy.

The Ethics Committee of IRCCS Istituto Oncologico “Gabriella Serio” approved the protocol cited for the present retrospective observational study (Protocol No. 1075, 17/12/2025, Prot. 1979/CEL—Studio L-PRF). The approval covers the retrospective analysis of clinical records obtained at both participating centers.

### 2.2. Surgically Assisted Expansion Protocol (Group 1)

- Pre-surgical orthodontic decompensation of the mandibular arch was performed to eliminate dentoalveolar compensations associated with the transverse maxillary deficiency.
- A fixed expander with a 12 mm central screw was connected by stainless-steel arms to orthodontic bands on teeth 16, 14, 24, and 26.
- Surgery consisted of a Le Fort I-type osteotomy. Bilateral full-thickness incisions were made from the distal aspect of the maxillary lateral incisor to the second premolar; after flap elevation, osteotomies were performed from the piriform aperture to the pterygoid processes, followed by pterygomaxillary disjunction and flap closure. A midline incision was then made between the maxillary central incisors from the vestibular fornix to the retroincisive papilla, followed by a vertical osteotomy through the midpalatal suture. The suture was completed with a chisel. The expander was activated intraoperatively by two full turns (2 mm), and an occlusal radiograph was obtained for control. On the first postoperative day, a further two quarter-turns were performed; thereafter, two quarter-turns were prescribed in the morning and two in the evening, until the planned expansion was achieved.
- The expansion screw was then secured with a ligature wire and the appliance was maintained in place for 8 months as retention.
- The expander was removed at the end of the retention period.

### 2.3. Orthopedic Expansion Protocol (Group 2)

- Rapid palatal expansion was performed using a 12 mm central expansion screw connected by stainless-steel arms to orthodontic bands. In the primary dentition, bands were cemented on teeth 55 and 65 with palatal extensions to teeth 53 and 63; in the mixed dentition, bands were placed on teeth 16 and 26 with similar palatal extensions.
- The expansion screw was activated by 0.5 mm/day, divided into two daily activations (morning and evening), until the planned expansion was achieved. The total amount of expansion was individualized, to obtain adequate overcorrection of the initial transverse discrepancy.
- The expansion screw was then secured with a ligature wire and the appliance was left in place for 6 months as retention.
- The expander was removed at the end of the retention period.

All patients were treated under the same operator’s clinical supervision using the cohort-specific protocol described above; no operator-specific variations in activation or retention instructions were planned. Appliance design principles and activation/retention procedures were standardized within each cohort; in the orthopedic cohort, anchorage was adapted to dentition stage, as described above. Clinical documentation included study casts and intraoral photographs obtained at baseline (T0), at T1, and, when available, at T2. Cephalometric assessment was based on lateral and posteroanterior cephalometric radiographs obtained at T0, immediately after appliance removal at T1, and at T2, 12 months after appliance removal (T1), when available. Radiographs were acquired with the patient upright in a cephalostat, with the head oriented according to the Frankfort horizontal plane and the dental arches in maximum intercuspation, using a standardized acquisition geometry with a focus-to-film distance of 1.50 m. Recruitment occurred at two centers within a shared clinical and training framework, with common operator supervision and standardized cohort-specific protocols. Although the clinical protocol and acquisition geometry were standardized, minor center-related differences in imaging equipment, appliance laboratory procedures, or follow-up logistics cannot be excluded, and represent a potential source of variability.

### 2.4. Records and Outcome Measurements

Digital cephalograms were traced by a single operator (L.G.) using Dolphin Imaging software v.11.1 (Dolphin Imaging and Management Solutions; Los Angeles, CA, USA), which calculated all reported measurements.

Each subject was assigned a random identification number, and the observer was blinded to subject identity during tracing. Fifteen randomly selected study records were re-evaluated for reproducibility: lateral and posteroanterior cephalograms were independently assessed by a second senior clinician (C.M.) and retraced by the first operator (L.G.) after 15 days; the corresponding dental-cast measurements were also repeated for reliability assessment. Quantitative reliability was evaluated using intraclass correlation coefficients (ICCs), with 95% confidence intervals, and is reported for each variable in Supplementary Table S1. For cephalometric measurements, intra-operator ICCs ranged from 0.889 to 0.972 and inter-operator ICCs from 0.842 to 0.955. For dental-cast measurements, intra-operator ICCs ranged from 0.962 to 0.986 and inter-operator ICCs from 0.941 to 0.975. Both examiners (L.G. and C.M.) were trained and experienced in cephalometric analyses, with prior research publications involving cephalometric measurements. Measurement reproducibility in the present study was objectively evaluated through the repeated-measurement protocol and the ICCs reported above.

Measurements were selected a priori to capture the principal anatomical domains potentially affected by maxillary expansion. Transverse variables quantified skeletal widening at the nasal, maxillary, mandibular, and molar levels and permitted side-specific assessment relative to the midline; SNA, SNB, and ANB summarized sagittal maxillomandibular relationships; and the selected vertical linear and angular measurements described maxillary inclination, mandibular rotation, and anterior facial height. Dental-cast measurements complemented cephalometry by quantifying arch-width and palatal-depth changes. The following variables were evaluated. To avoid ambiguity, in the context of skeletal maturity, CVM denotes cervical vertebral maturation; the cephalometric labels CVM+ and CVM- used below refer to molar landmarks, and are unrelated to the maturation assessment.

Transverse relationships (posteroanterior cephalogram): piriform aperture width (nasal cavity width); MXR-MXL (distance between the right and left maxillary points); GOR-GOL (intergonial width); CVM+R-CVM+L (distance between the right and left maxillary molar points); CVM-R-CVM-L (distance between the right and left mandibular molar points); and the distance of each right (R) and left (L) landmark (MX, GO, CVM+, and CVM-) from the midline symmetry axis.

Sagittal relationships (lateral cephalogram): SNA, SNB, and ANB angles.

Vertical relationships (lateral cephalogram): SOR-SNA (upper anterior skeletal vertical dimension); SNA-Me (lower anterior skeletal vertical dimension); SOR-Me (total anterior skeletal vertical dimension); SN^SNP-SNA (cranio–spinal angle, reflecting maxillary inclination relative to the cranial base); and SN^Go-Gn (cranio–mandibular angle, reflecting mandibular inclination relative to the cranial base).

**Figure 1:**
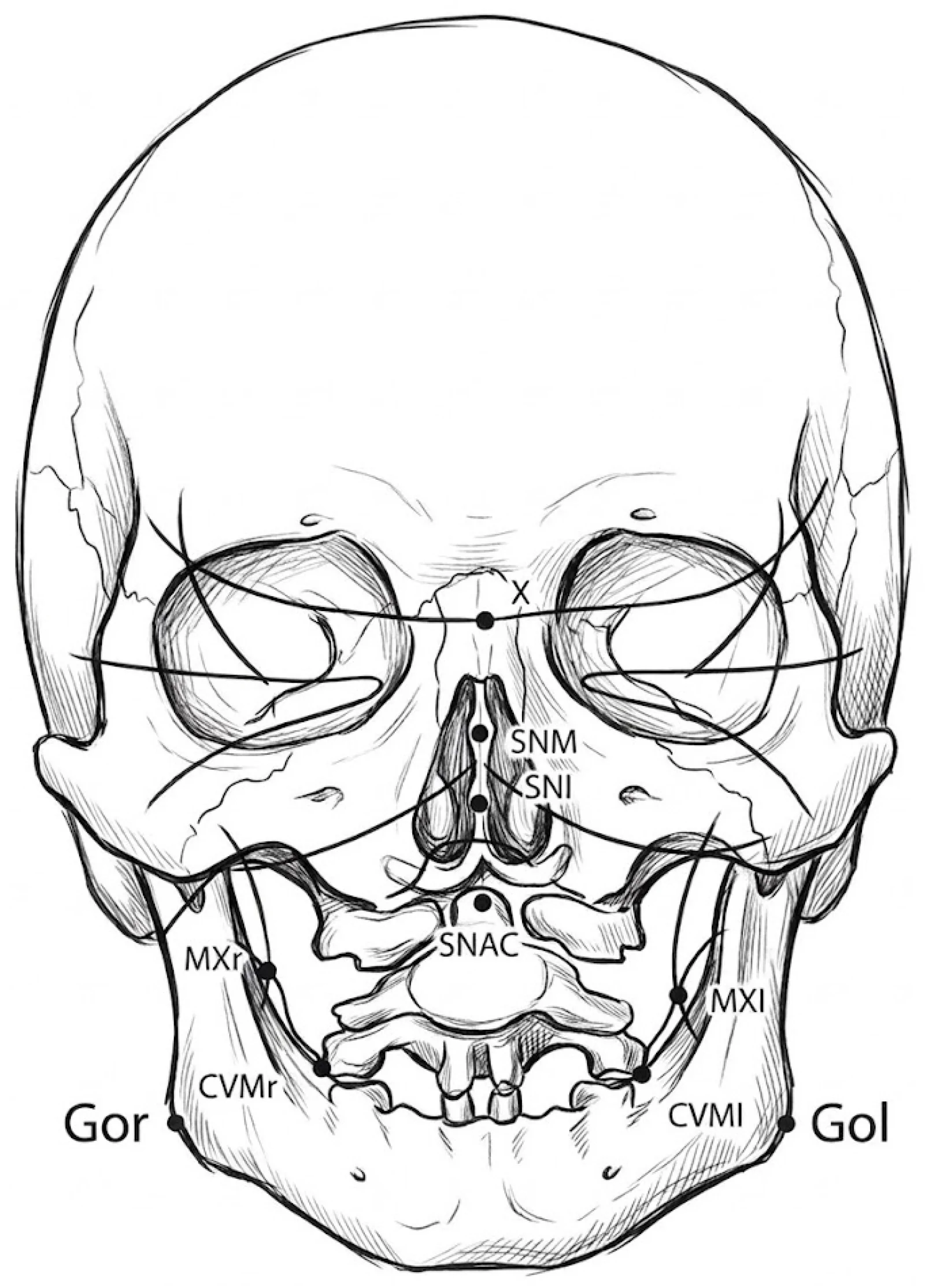
Posteroanterior cephalometric landmarks and reference points relevant to the transverse analysis. R = right; L = left.

The main posteroanterior cephalometric landmarks and reference points used for the transverse measurements are illustrated in Figure 1.

Accordingly, the present assessment is described as multiplanar, rather than three-dimensional: it integrates lateral and posteroanterior two-dimensional cephalometric projections with dental-cast measurements, and no volumetric CBCT-based analysis was performed.

Baseline (T0) lateral and posteroanterior cephalograms were obtained no more than two months before appliance cementation. Post-treatment (T1) cephalograms were obtained immediately after appliance removal, approximately 9–12 months after treatment initiation. The appliance-retention phase lasted 8 months in the surgically assisted cohort and 6 months in the orthopedic cohort, as specified in Section 2.2 and Section 2.3; thus, T1 represents the assessment immediately after completion of treatment with appliance removal. In both cohorts, T2 assessments were performed 12 months after appliance removal (T1) for patients with available follow-up records. Differences in retention duration and total treatment exposure may have influenced the magnitude of the observed T0–T1 changes, and should be considered when contextualizing the two cohorts.

Dental casts obtained at T0, T1, and, when available, T2, were used to measure maxillary and mandibular intercanine and intermolar widths. When the permanent first molars had not erupted, the second primary molars were used. Mandibular measurements served as a reference for the amount of maxillary expansion. Intermolar width was measured between the central fossae of the first molars, intercanine width between canine cusp tips, and palatal depth from the deepest point of the palatal vault to a ruler positioned at the occlusal level of the molars and premolars. Palatal rugae were used as reference landmarks for repeated measurements.

Because transverse skeletal measurements were derived from posteroanterior cephalograms, they remain susceptible to two-dimensional projection limitations, including sensitivity to head positioning, magnification, landmark superimposition, and projection geometry. Standardized cephalostat positioning and acquisition geometry were used to reduce these sources of error, but they cannot be eliminated as they could with volumetric imaging.

### 2.5. Statistical Analysis

Sample size calculation. Because the study was designed to estimate within-cohort T0–T1 changes, rather than to test between-cohort hypotheses, sample size was planned a priori on the precision of estimation, rather than on statistical power. Precision was defined as the half-width of the two-sided 95% confidence interval for the mean T0–T1 change, expressed relative to the underlying population standard deviation (SD) of individual change scores, thereby providing a common criterion across continuous outcomes. A maximum half-width of 0.50 SD was prespecified as the acceptable precision criterion. To account for uncertainty in the estimated SD, the calculation incorporated an assurance probability of 80% under normality and used the exact Student t and chi-square distributions. This yielded a minimum of 22 analyzable patients per cohort. Allowing for approximately 10% of records to be unusable, at least 25 potentially eligible records per cohort were targeted for retrieval. The calculation was applied separately to each cohort, consistent with their analysis as parallel descriptive populations, and no between-cohort comparison was planned. Because 22 represented the minimum analyzable sample, all consecutive eligible patients treated during the January 2024–December 2025 inclusion period were included, yielding 27 surgically assisted and 54 orthopedic patients. T2 follow-up analyses were not included in the sample-size planning, and were considered exploratory. T2 follow-up records were available for 18 surgically assisted and 20 orthopedic patients. Follow-up analyses were performed on an available-case basis, and missing follow-up values were not imputed. T0–T1 change was defined as T1 minus T0, whereas follow-up change was defined as T2 minus T1. The distribution of change scores was assessed descriptively, through the joint presentation of mean and SD, median, and interquartile range; because the analysis was descriptive, and T2 was not part of the a priori sample-size planning, no formal normality-testing or normality-driven inferential strategy was used. Two-sided 95% confidence intervals for mean change were calculated using the Student t distribution when SD was available. No null-hypothesis significance tests were performed and, consequently, no alpha level was adopted and no multiplicity adjustment was applied. The cohorts represented different clinical populations defined by age, growth status, and treatment indication; therefore, no confirmatory between-group hypothesis testing, equivalence testing, or causal comparison of treatment effectiveness was undertaken. Interpretation was based on the magnitude, direction, precision, and internal consistency of observed within-cohort changes. Missing T2 data may introduce attrition bias if patients with available records differ systematically from those without follow-up.

## 3. Results

### 3.1. Cohort Characteristics and Follow-Up Availability

Eighty-one consecutive patients met eligibility criteria and had analyzable T0 and T1 records: 54 in the orthopedic cohort and 27 in the surgically assisted cohort. All 81 contributed to the primary T0–T1 analyses. T2 assessments were performed 12 months after appliance removal (T1) in patients with available follow-up records; T2 cephalometric records were available for 20/54 orthopedic patients (37.0%) and 18/27 surgical patients (66.7%), while dental-cast T2 records were available for 18 patients in each cohort (33.3% and 66.7%, respectively). The 18 orthopedic patients with available T2 casts were a subset of the 20 with T2 cephalograms, and the same 18 surgical patients contributed both modalities, as documented in the table notes. Because follow-up availability may not have been random, the available-case estimates may be affected by attrition bias. T1–T2 observations are reported separately, and should not be interpreted as definitive evidence of stability, regression, or relapse.

### 3.2. Sagittal Relationships (Table 2 and Table 3)

In the orthopedic cohort, T0–T1 sagittal changes were modest. SNA increased by 0.89 ± 1.59° (95% CI 0.46 to 1.32), SNB changed by −0.46 ± 1.65° (95% CI −0.91 to −0.01), and ANB increased by 1.35 ± 1.47° (95% CI 0.95 to 1.75). Among the 20 patients with cephalometric follow-up, mean T2–T1 changes were −0.10° for SNA (95% CI −0.87 to 0.67), +0.93° for SNB (0.33 to 1.53), and −1.03° for ANB (−1.80 to −0.26). These available-case estimates cannot establish long-term stability or treatment-related reversal. In this growing cohort, the absence of an untreated control additionally prevents separation of treatment-associated effects from maturation.

In the surgically assisted cohort, T0–T1 mean changes were −1.05 ± 2.57° for SNA (95% CI −2.07 to −0.03), −1.43 ± 2.10° for SNB (−2.26 to −0.60), and +0.38 ± 1.97° for ANB (−0.40 to 1.16). At T1–T2 (*n* = 18), mean changes were +0.77 ± 1.50° for SNA (95% CI 0.02 to 1.52), +1.22 ± 0.96° for SNB (0.74 to 1.70), and −0.45 ± 1.25° for ANB (−1.07 to 0.17). The corresponding mean ANB changes were internally consistent with SNA minus SNB. Follow-up results remain exploratory because of incomplete records and potential attrition bias.

Within the subset for which skeletal-class classification was available, the qualitative pattern previously observed was maintained: Class III subjects tended to show an increase in ANB, whereas changes in Class I and Class II were less uniform. Because these subgroup observations were based on small numbers and were not supported by separate inferential analyses, they should be regarded as exploratory.

**Table 2:** Changes in sagittal cephalometric relationships in orthopedically treated patients.

Variable	Interval	*n*	Mean ± SD [95% CI]	Median [Q1, Q3]
SNA	T0–T1	54	0.89 ± 1.59 [0.46, 1.32]	0.73 [−0.18, 1.72]
SNA	Follow-up	20	−0.10 ± 1.65 [−0.87, 0.67]	0.14 [−1.26, 0.96]
SNB	T0–T1	54	−0.46 ± 1.65 [−0.91, −0.01]	−0.50 [−1.70, 0.59]
SNB	Follow-up	20	0.93 ± 1.28 [0.33, 1.53]	0.64 [0.09, 1.96]
ANB	T0–T1	54	1.35 ± 1.47 [0.95, 1.75]	1.64 [0.57, 2.43]
ANB	Follow-up	20	−1.03 ± 1.65 [−1.80, −0.26]	−1.16 [−2.44, 0.03]

T0–T1 = change from baseline (T0) to the post-treatment assessment (T1); follow-up = subsequent change from T1 to T2, scheduled approximately 1 year after appliance removal. Angular values are in degrees. *n* = number of patients with paired measurements available for each interval; T2 records were available for 20 of 54 patients (37.0%), and 34 patients (63.0%) had no available T2 records. Follow-up estimates are exploratory, because T2 was not included in the a priori sample-size planning and longitudinal data were incomplete.

**Table 3:** Changes in sagittal cephalometric relationships in surgically treated patients.

Variable	Interval	*n*	Mean ± SD [95% CI]	Median [Q1, Q3]
SNA	T0–T1	27	−1.05 ± 2.57 [−2.07, −0.03]	−1.46 [−2.88, 0.77]
SNA	Follow-up	18	0.77 ± 1.50 [0.02, 1.52]	1.08 [−0.01, 1.94]
SNB	T0–T1	27	−1.43 ± 2.10 [−2.26, −0.60]	−1.08 [−2.91, −0.02]
SNB	Follow-up	18	1.22 ± 0.96 [0.74, 1.70]	1.29 [0.63, 1.92]
ANB	T0–T1	27	0.38 ± 1.97 [−0.40, 1.16]	0.21 [−0.57, 1.82]
ANB	Follow-up	18	−0.45 ± 1.25 [−1.07, 0.17]	−0.17 [−1.53, 0.32]

T0–T1 = change from baseline (T0) to the post-treatment assessment (T1); follow-up = subsequent change from T1 to T2, scheduled approximately 1 year after appliance removal. Angular values are in degrees. *n* = number of patients with paired measurements available for each interval; T2 records were available for 18 of 27 patients (66.7%), and 9 patients (33.3%) had no available T2 records. Values are mean ± SD [95% CI] and median [Q1, Q3]. Follow-up estimates are exploratory, because T2 was not included in the a priori sample-size planning and longitudinal data were incomplete.

### 3.3. Vertical Relationships (Table 4 and Table 5)

In the orthopedic cohort, T0–T1 vertical changes were generally small, relative to their variability. SOR–SNA changed by +0.03 ± 0.95 mm (95% CI −0.23 to 0.29), while SNA–Me increased by 1.01 ± 2.51 mm (0.32 to 1.70), SOR–Me by 1.04 ± 2.55 mm (0.34 to 1.74), and SN^Go–Gn by 0.86 ± 2.49° (0.18 to 1.54). At T1–T2 (*n* = 20), mean changes were +0.60 mm for SOR–SNA (0.09 to 1.11), +0.45 mm for SNA–Me (−0.70 to 1.60), +1.05 mm for SOR–Me (−0.13 to 2.23), and −0.94° for SN^Go–Gn (−2.03 to 0.15). The vertical linear means are consistent with the stated additive landmark convention. Follow-up findings are descriptive, and do not establish stability or reversal, particularly without an untreated pediatric growth control.

In the surgically assisted cohort, T0–T1 mean changes included +1.68 ± 2.30 mm for SNA–Me (95% CI 0.77 to 2.59), +1.76 ± 2.41 mm for SOR–Me (0.81 to 2.71), −0.07 ± 1.15° for SN^SNP–SNA (−0.52 to 0.38), and +1.58 ± 2.03° for SN^Go–Gn (0.78 to 2.38). At T1–T2 (*n* = 18), SNA–Me changed by −1.66 mm (−2.59 to −0.73), SOR–Me by −1.23 mm (−3.11 to 0.65), and SN^Go–Gn by −1.48° (−1.87 to −1.09). These estimates remain descriptive, and should not be interpreted as proof of stability, relapse, or persistent skeletal effects.

Descriptions by normodivergent, skeletal open-bite, or skeletal deep-bite morphology remain exploratory because of the small subgroup sizes.

**Table 4:** Changes in vertical cephalometric relationships in orthopedically treated patients.

Variable	Interval	*n*	Mean ± SD [95% CI]	Median [Q1, Q3]
SOR-SNA	T0–T1	54	0.03 ± 0.95 [−0.23, 0.29]	0.01 [−0.63, 0.77]
SOR-SNA	Follow-up	20	0.60 ± 1.10 [0.09, 1.11]	0.50 [−0.11, 1.30]
SNA-Me	T0–T1	54	1.01 ± 2.51 [0.32, 1.70]	1.13 [−0.88, 2.83]
SNA-Me	Follow-up	20	0.45 ± 2.45 [−0.70, 1.60]	1.02 [−1.08, 2.50]
SOR-Me	T0–T1	54	1.04 ± 2.55 [0.34, 1.74]	0.87 [−0.43, 2.77]
SOR-Me	Follow-up	20	1.05 ± 2.53 [−0.13, 2.23]	1.20 [−0.62, 2.42]
SN^SNP-SNA	T0–T1	54	−0.03 ± 1.27 [−0.38, 0.32]	−0.09 [−0.95, 0.86]
SN^SNP-SNA	Follow-up	20	−0.47 ± 1.25 [−1.06, 0.12]	−0.35 [−1.07, 0.38]
SN^Go-Gn	T0–T1	54	0.86 ± 2.49 [0.18, 1.54]	0.41 [−0.83, 2.41]
SN^Go-Gn	Follow-up	20	−0.94 ± 2.33 [−2.03, 0.15]	−0.98 [−2.80, 0.43]

T0–T1 = change from baseline (T0) to the post-treatment assessment (T1); follow-up = subsequent change from T1 to T2, scheduled approximately 1 year after appliance removal. Linear measurements are in millimeters and angular measurements in degrees. *n* = number of patients with paired measurements available for each interval; T2 records were available for 20 of 54 patients (37.0%), and 34 patients (63.0%) had no available T2 records. Vertical distances are measured along the same reference line, so that SOR-Me = SOR-SNA + SNA-Me for each patient. Values are mean ± SD [95% CI] and median [Q1, Q3]. Follow-up estimates are exploratory, because T2 was not included in the a priori sample-size planning and longitudinal data were incomplete.

**Table 5:** Changes in vertical cephalometric relationships in surgically treated patients.

Variable	Interval	*n*	Mean ± SD [95% CI]	Median [Q1, Q3]
SOR-SNA	T0–T1	27	0.08 ± 1.83 [−0.64, 0.80]	0.40 [−1.10, 1.60]
SOR-SNA	Follow-up	18	0.43 ± 2.50 [−0.81, 1.67]	0.07 [−1.16, 2.26]
SNA-Me	T0–T1	27	1.68 ± 2.30 [0.77, 2.59]	2.12 [0.11, 3.32]
SNA-Me	Follow-up	18	−1.66 ± 1.87 [−2.59, −0.73]	−2.08 [−3.09, −0.53]
SOR-Me	T0–T1	27	1.76 ± 2.41 [0.81, 2.71]	1.93 [0.34, 3.65]
SOR-Me	Follow-up	18	−1.23 ± 3.79 [−3.11, 0.65]	−1.04 [−3.75, 1.72]
SN^SNP-SNA	T0–T1	27	−0.07 ± 1.15 [−0.52, 0.38]	0.20 [−0.74, 0.80]
SN^SNP-SNA	Follow-up	18	−0.37 ± 1.05 [−0.89, 0.15]	−0.60 [−1.06, 0.41]
SN^Go-Gn	T0–T1	27	1.58 ± 2.03 [0.78, 2.38]	1.90 [0.37, 3.27]
SN^Go-Gn	Follow-up	18	−1.48 ± 0.78 [−1.87, −1.09]	−1.61 [−1.99, −1.07]

T0–T1 = change from baseline (T0) to the post-treatment assessment (T1); follow-up = subsequent change from T1 to T2, scheduled approximately 1 year after appliance removal. Linear measurements are in millimeters and angular measurements in degrees. *n* = number of patients with paired measurements available for each interval; T2 records were available for 18 of 27 patients (66.7%), and 9 patients (33.3%) had no available T2 records. Vertical distances are measured along the same reference line, so that SOR-Me = SOR-SNA + SNA-Me for each patient. Values are mean ± SD [95% CI] and median [Q1, Q3]. Follow-up estimates are exploratory, because T2 was not included in the a priori sample-size planning and longitudinal data were incomplete.

### 3.4. Transverse Relationships (Table 6, Table 7 and Table 8)

Transverse T0–T1 changes were more pronounced than most sagittal and vertical changes. In the orthopedic cohort, piriform aperture width increased by 1.72 ± 1.45 mm (95% CI 1.32 to 2.12), MXR–MXL by 3.56 ± 2.62 mm (2.84 to 4.28), and cephalometric maxillary intermolar width (CVM+R–CVM+L) by 6.56 ± 2.81 mm (5.79 to 7.33). Mandibular intermolar width increased by 0.82 ± 1.50 mm (0.41 to 1.23), whereas intergonial width changed by +0.17 mm (−0.02 to 0.36). Among the 20 patients with T1–T2 cephalograms, MXR–MXL changed by +0.43 ± 1.06 mm (−0.07 to 0.93), upper-intermolar width by +0.42 ± 1.18 mm (−0.13 to 0.97), and lower-intermolar width by −0.24 ± 0.66 mm (−0.55 to 0.07). Because the follow-up sample was incomplete, these observations do not establish treatment stability or relapse.

In the surgically assisted cohort, T0–T1 piriform aperture width increased by 1.17 ± 0.75 mm (95% CI 0.87 to 1.47), MXR–MXL by 1.39 ± 1.60 mm (0.76 to 2.02), and cephalometric maxillary intermolar width by 5.43 ± 1.21 mm (4.95 to 5.91). At T1–T2 (*n* = 18), MXR–MXL changed by −0.36 ± 0.87 mm (−0.79 to 0.07), maxillary intermolar width by −0.38 ± 0.84 mm (−0.80 to 0.04), and mandibular intermolar width by −0.27 ± 0.89 mm (−0.71 to 0.17). These small follow-up changes, with confidence intervals spanning zero, are descriptive, and do not establish long-term stability or relapse in the presence of incomplete patient-level follow-up.

Side-specific analysis of the surgically treated sample showed mean T0–T1 increases of +2.52 mm on the right and +2.91 mm on the left for the upper-molar landmarks (CVM+R and CVM+L), with 95% CIs of 2.23 to 2.81 and 2.61 to 3.21 mm, respectively. Their sum corresponds to the +5.43 mm bilateral width change. At T1–T2, the respective means were −0.17 and −0.21 mm, corresponding to the bilateral change of −0.38 mm. Side-specific changes at other landmarks were variable; in the absence of a prespecified asymmetry outcome and patient-level analysis, no specific mechanism of asymmetry correction can be inferred.

**Table 6:** Changes in transverse cephalometric relationships in orthopedically treated patients.

Variable	Interval	*n*	Mean ± SD [95% CI]	Median [Q1, Q3]
Piriform	T0–T1	54	1.72 ± 1.45 [1.32, 2.12]	1.53 [0.64, 2.60]
Piriform	Follow-up	20	0.43 ± 1.91 [−0.46, 1.32]	0.77 [−0.75, 1.88]
MXR-MXL	T0–T1	54	3.56 ± 2.62 [2.84, 4.28]	2.92 [1.74, 5.21]
MXR-MXL	Follow-up	20	0.43 ± 1.06 [−0.07, 0.93]	0.32 [−0.44, 1.05]
CVM+R-CVM+L	T0–T1	54	6.56 ± 2.81 [5.79, 7.33]	6.84 [4.95, 8.28]
CVM+R-CVM+L	Follow-up	20	0.42 ± 1.18 [−0.13, 0.97]	0.45 [−0.45, 1.05]
CVM-R-CVM-L	T0–T1	54	0.82 ± 1.50 [0.41, 1.23]	0.55 [−0.18, 1.78]
CVM-R-CVM-L	Follow-up	20	−0.24 ± 0.66 [−0.55, 0.07]	−0.32 [−0.67, 0.14]
GOR-GOL	T0–T1	54	0.17 ± 0.71 [−0.02, 0.36]	0.05 [−0.35, 0.66]
GOR-GOL	Follow-up	20	1.20 ± 0.95 [0.76, 1.64]	1.05 [0.50, 1.87]

T0–T1 = change from baseline (T0) to the post-treatment assessment (T1); follow-up = subsequent change from T1 to T2, scheduled approximately 1 year after appliance removal. All values are in millimeters. *n* = number of patients with paired measurements available for each interval; T2 records were available for 20 of 54 patients (37.0%), and 34 patients (63.0%) had no available T2 records. Values are mean ± SD [95% CI] and median [Q1, Q3]. Follow-up estimates are exploratory, because T2 was not included in the a priori sample-size planning and longitudinal data were incomplete.

**Table 7:** Changes in transverse cephalometric relationships in surgically treated patients.

Variable	Interval	*n*	Mean ± SD [95% CI]	Median [Q1, Q3]
Piriform	T0–T1	27	1.17 ± 0.75 [0.87, 1.47]	1.07 [0.67, 1.72]
Piriform	Follow-up	18	−0.15 ± 0.85 [−0.57, 0.27]	−0.18 [−0.53, 0.59]
MXR-MXL	T0–T1	27	1.39 ± 1.60 [0.76, 2.02]	1.25 [0.30, 2.21]
MXR-MXL	Follow-up	18	−0.36 ± 0.87 [−0.79, 0.07]	−0.43 [−0.86, 0.23]
CVM+R-CVM+L	T0–T1	27	5.43 ± 1.21 [4.95, 5.91]	5.53 [4.76, 6.29]
CVM+R-CVM+L	Follow-up	18	−0.38 ± 0.84 [−0.80, 0.04]	−0.24 [−0.77, 0.25]
CVM-R-CVM-L	T0–T1	27	0.50 ± 2.27 [−0.40, 1.40]	0.23 [−1.33, 1.80]
CVM-R-CVM-L	Follow-up	18	−0.27 ± 0.89 [−0.71, 0.17]	−0.14 [−0.86, 0.20]
GOR-GOL	T0–T1	27	0.08 ± 1.24 [−0.41, 0.57]	−0.02 [−0.67, 0.86]
GOR-GOL	Follow-up	18	0.29 ± 2.67 [−1.04, 1.62]	0.36 [−1.34, 1.96]

T0–T1 = change from baseline (T0) to the post-treatment assessment (T1); follow-up = subsequent change from T1 to T2, scheduled approximately 1 year after appliance removal. All values are in millimeters. *n* = number of patients with paired measurements available for each interval; T2 records were available for 18 of 27 patients (66.7%), and 9 patients (33.3%) had no available T2 records. The same 18 surgical patients contributed T2 cephalometric and dental-cast measurements, as detailed in the corresponding tables. Values are mean ± SD [95% CI] and median [Q1, Q3]. Follow-up estimates are exploratory, because T2 was not included in the a priori sample-size planning and longitudinal data were incomplete.

**Table 8:** Side-specific transverse changes relative to the midline symmetry axis in surgically treated patients.

Variable	Interval	*n*	Mean ± SD [95% CI]	Median [Q1, Q3]
MXR	T0–T1	27	0.97 ± 0.89 [0.62, 1.32]	0.86 [0.50, 1.58]
MXR	Follow-up	18	−0.21 ± 0.62 [−0.52, 0.10]	−0.18 [−0.56, 0.21]
MXL	T0–T1	27	0.42 ± 1.09 [−0.01, 0.85]	0.21 [−0.45, 0.87]
MXL	Follow-up	18	−0.15 ± 0.46 [−0.38, 0.08]	−0.16 [−0.48, 0.14]
CVM+R	T0–T1	27	2.52 ± 0.74 [2.23, 2.81]	2.69 [2.06, 2.96]
CVM+R	Follow-up	18	−0.17 ± 0.43 [−0.38, 0.04]	−0.12 [−0.43, 0.15]
CVM+L	T0–T1	27	2.91 ± 0.75 [2.61, 3.21]	2.95 [2.55, 3.47]
CVM+L	Follow-up	18	−0.21 ± 0.61 [−0.51, 0.09]	−0.22 [−0.52, 0.29]
CVM-R	T0–T1	27	0.03 ± 0.94 [−0.34, 0.40]	0.19 [−0.70, 0.59]
CVM-R	Follow-up	18	−0.08 ± 0.61 [−0.38, 0.22]	−0.03 [−0.47, 0.28]
CVM-L	T0–T1	27	0.47 ± 1.79 [−0.24, 1.18]	0.26 [−0.72, 1.38]
CVM-L	Follow-up	18	−0.19 ± 0.50 [−0.44, 0.06]	−0.20 [−0.62, 0.06]
GOR	T0–T1	27	0.05 ± 0.64 [−0.20, 0.30]	−0.02 [−0.47, 0.45]
GOR	Follow-up	18	0.18 ± 1.80 [−0.72, 1.08]	0.27 [−1.09, 1.35]
GOL	T0–T1	27	0.03 ± 0.85 [−0.31, 0.37]	0.09 [−0.48, 0.56]
GOL	Follow-up	18	0.11 ± 1.47 [−0.62, 0.84]	0.01 [−0.82, 1.06]

T0–T1 = change from baseline (T0) to the post-treatment assessment (T1); follow-up = subsequent change from T1 to T2, scheduled approximately 1 year after appliance removal. All values are in millimeters. *n* = number of patients with paired measurements available for each interval; T2 records were available for 18 of 27 patients (66.7%), and 9 patients (33.3%) had no available T2 records. Side-specific distances are measured from the midline symmetry axis to the right (R) and left (L) landmarks; for each patient, the right plus left change equals the corresponding bilateral width change reported in Table 7. Values are mean ± SD [95% CI] and median [Q1, Q3]. Follow-up estimates are exploratory, because T2 was not included in the a priori sample-size planning and longitudinal data were incomplete.

### 3.5. Dental Cast Measurements (Table 9 and Table 10)

Dental-cast measurements showed T0–T1 maxillary widening in the orthopedic cohort. Maxillary intercanine width (IC+) increased by 2.86 ± 2.41 mm (95% CI 2.20 to 3.52) and maxillary intermolar width (IM+) by 6.83 ± 2.79 mm (6.07 to 7.59); palatal depth decreased by −1.21 ± 0.99 mm (−1.48 to −0.94). Mandibular intercanine width (IC−) increased by 1.57 ± 0.79 mm (1.35 to 1.79), and mandibular intermolar width (IM−) by 0.87 ± 1.04 mm (0.59 to 1.15). Among the 18 patients with T2 casts, T1–T2 upper-intercanine width changed by +1.38 ± 1.46 mm (0.65 to 2.11) and upper-intermolar width by +0.44 ± 1.12 mm (−0.12 to 1.00). These follow-up results remain exploratory.

In the surgically assisted cohort, T0–T1 maxillary intercanine and intermolar changes were +5.60 ± 2.97 mm (95% CI 4.43 to 6.77) and +5.71 ± 1.69 mm (5.04 to 6.38), respectively, while palatal depth decreased by −1.60 ± 1.14 mm (−2.05 to −1.15). Among the same 18 surgical patients with T2 cephalometric and cast records, T1–T2 maxillary intercanine width changed by −1.14 ± 1.35 mm (−1.81 to −0.47) and maxillary intermolar width by −0.41 ± 0.79 mm (−0.80 to −0.02). The T1–T2 values describe the available sample only; incomplete ascertainment and the lack of a prespecified confirmatory T2 analysis prevent definitive inferences regarding relapse or stability.

**Table 9:** Dental-cast changes in orthopedically treated patients.

Variable	Interval	*n*	Mean ± SD [95% CI]	Median [Q1, Q3]
IC+	T0–T1	54	2.86 ± 2.41 [2.20, 3.52]	2.66 [1.19, 4.15]
IC+	Follow-up	18	1.38 ± 1.46 [0.65, 2.11]	1.59 [0.28, 2.05]
IM+	T0–T1	54	6.83 ± 2.79 [6.07, 7.59]	6.90 [5.00, 9.03]
IM+	Follow-up	18	0.44 ± 1.12 [−0.12, 1.00]	0.56 [−0.37, 1.14]
Palatal depth	T0–T1	54	−1.21 ± 0.99 [−1.48, −0.94]	−1.04 [−1.66, −0.49]
Palatal depth	Follow-up	18	0.10 ± 1.12 [−0.46, 0.66]	−0.02 [−0.74, 0.71]
IC-	T0–T1	54	1.57 ± 0.79 [1.35, 1.79]	1.41 [0.93, 2.02]
IC-	Follow-up	18	0.33 ± 0.58 [0.04, 0.62]	0.29 [−0.18, 0.63]
IM-	T0–T1	54	0.87 ± 1.04 [0.59, 1.15]	0.83 [0.02, 1.58]
IM-	Follow-up	18	−0.26 ± 0.61 [−0.56, 0.04]	−0.18 [−0.59, 0.17]

IC+ = maxillary intercanine width; IM+ = maxillary intermolar width; IC- = mandibular intercanine width; IM- = mandibular intermolar width. T0–T1 = change from baseline (T0) to the post-treatment assessment (T1); follow-up = subsequent change from T1 to T2, scheduled approximately 1 year after appliance removal. All values are in millimeters. *n* = number of patients with paired measurements available for each interval; T2 records were available for 18 of 54 patients (33.3%), and 36 patients (66.7%) had no available T2 records. The 18 patients with T2 dental casts are a subset of the 20 orthopedic patients with T2 cephalograms (Table 2, Table 4 and Table 6). Follow-up estimates are exploratory, because T2 was not included in the a priori sample-size planning and longitudinal data were incomplete.

**Table 10:** Dental-cast changes in surgically treated patients.

Variable	Interval	*n*	Mean ± SD [95% CI]	Median [Q1, Q3]
IC+	T0–T1	27	5.60 ± 2.97 [4.43, 6.77]	5.89 [3.75, 7.87]
IC+	Follow-up	18	−1.14 ± 1.35 [−1.81, −0.47]	−1.32 [−1.95, −0.26]
IM+	T0–T1	27	5.71 ± 1.69 [5.04, 6.38]	6.01 [4.73, 6.82]
IM+	Follow-up	18	−0.41 ± 0.79 [−0.80, −0.02]	−0.52 [−0.99, 0.03]
Palatal depth	T0–T1	27	−1.60 ± 1.14 [−2.05, −1.15]	−1.88 [−2.33, −0.87]
Palatal depth	Follow-up	18	0.21 ± 0.96 [−0.27, 0.69]	0.12 [−0.57, 0.82]
IC-	T0–T1	27	0.03 ± 1.63 [−0.61, 0.67]	−0.04 [−1.11, 1.27]
IC-	Follow-up	18	0.27 ± 0.49 [0.03, 0.51]	0.22 [−0.09, 0.48]
IM-	T0–T1	27	0.53 ± 2.35 [−0.40, 1.46]	0.33 [−1.31, 1.85]
IM-	Follow-up	18	−0.29 ± 0.61 [−0.59, 0.01]	−0.15 [−0.66, 0.24]

IC+ = maxillary intercanine width; IM+ = maxillary intermolar width; IC- = mandibular intercanine width; IM- = mandibular intermolar width. T0–T1 = change from baseline (T0) to the post-treatment assessment (T1); follow-up = subsequent change from T1 to T2, scheduled approximately 1 year after appliance removal. All values are in millimeters. *n* = number of patients with paired measurements available for each interval; T2 records were available for 18 of 27 patients (66.7%), and 9 patients (33.3%) had no available T2 records. The same 18 surgical patients contributed T2 cephalometric (Table 3, Table 5, Table 7 and Table 8) and dental-cast data (Table 10). Follow-up estimates are exploratory, because T2 was not included in the a priori sample-size planning and longitudinal data were incomplete.

## 4. Discussion

The principal finding was transverse skeletal and dentoalveolar widening from T0 to T1 within each cohort, whereas sagittal and vertical changes were generally smaller and more variable. Orthopedic RPE was used in growing patients and surgically assisted expansion in skeletally mature adults. These parallel descriptive cohorts differ in growth status and indication; their outcome magnitudes cannot be interpreted as a direct comparison of treatment effects. In the pediatric cohort, the absence of an untreated growth control further limits attribution of changes to expansion alone.

The transverse findings are broadly consistent with the classical literature. Wertz [11] described skeletal and dental changes accompanying midpalatal suture opening. Zhao et al. [16] and Niu et al. [17] reviewed nasal-cavity and upper-airway outcomes of maxillary expansion, while Pirelli et al. [18] investigated skeletal and nasomaxillary volume changes in children with obstructive sleep apnea; these studies concern anatomical domains related to, but not identical to, the transverse measurements used here. The present data show larger changes at the maxillary dental level than at mandibular or intergonial landmarks. These findings are also in line with evidence that RME performed before the pubertal growth peak produces greater skeletal transverse effects and with systematic-review data indicating that long-term skeletal gains are more favorable in less skeletally mature patients [27,28]. Three-dimensional imaging studies have further characterized the relative skeletal and dentoalveolar components and regional heterogeneity of expansion [29,30]. The surgically assisted sample also demonstrated substantial maxillary intermolar widening, supporting the rationale for reducing mature circummaxillary resistance when a skeletal transverse correction is required in nongrowing patients. Reviews of SARME/SARPE support surgical assistance in skeletally mature patients, while emphasizing heterogeneity in surgical technique, appliance design, and expansion pattern [31,32]. At the same time, the wide interval around MXR-MXL in the surgical analytical sample indicates that the magnitude of skeletal widening was variable and should not be inferred solely from dental expansion.

Skeletal maturation is important for interpreting orthopedic expansion because sutural resistance and the relative skeletal and dentoalveolar contributions may change during growth. CVM staging was assessed in all patients; approximately 30%, 50%, and 20% of the orthopedic cohort were classified as CS2, CS3, and CS4, respectively. The wide 5–17-year age range and absence of stage-specific analyses or an untreated growth control prevent disentangling growth, maturation, and treatment-associated changes. The adult surgical cohort had a distinct clinical indication.

A broader biological perspective is also relevant. Maxillofacial bone differs from long bone in developmental origin, predominant ossification pathway, local cellular phenotype, architecture, and functional loading environment. The maxilla and mandible are largely neural-crest derived, and develop predominantly through intramembranous ossification, whereas long bones are mesoderm-derived, and typically form through endochondral ossification; site-specific skeletal progenitor behavior and remodeling characteristics may therefore influence regional adaptation [37]. In the context of maxillary expansion, these biological distinctions provide a plausible framework for heterogeneous responses across sutural, basal skeletal, alveolar, and dentoalveolar regions, although the present study did not measure cellular or molecular remodeling markers.

Sagittal changes were comparatively modest. In the orthopedic sample, the T0–T1 increase in ANB was accompanied by a small reduction in SNB. Among the available T2 observations, the mean ANB change was negative; however, because follow-up records were incomplete, this direction should not be interpreted as recovery, regression, or long-term stability. The T0–T1 pattern may reflect a combination of treatment-related occlusal/mandibular repositioning and normal craniofacial growth; without an untreated age- and maturation-matched control group, the relative contribution of these mechanisms cannot be separated. This interpretation is compatible with previous descriptions of post-expansion skeletal positional changes by Wertz [11] and with reports of sagittal effects in surgically assisted expansion by Chung et al. [23]; Gilon et al. [22] discussed surgically assisted maxillary disjunction in the broader orthodontic-surgical context. The class-related tendency toward increased ANB in Class III subjects is clinically interesting, and may be contextualized by reported sagittal effects across different malocclusion groups studied by Maspero et al. [24], but the present subgroup sizes are too small to support a class-specific treatment claim.

Vertical changes were generally small and heterogeneous. In the orthopedic cohort, the upper anterior (SOR–SNA), lower anterior (SNA–Me), and total anterior (SOR–Me) linear changes between T0 and T1 were +0.03, +1.01, and +1.04 mm, respectively, in accordance with their additive definition. Available T1–T2 changes in lower and total anterior dimensions were also positive (+0.45 and +1.05 mm), while the cranio–mandibular angle changed by −0.94°. This pattern cannot establish reversal, recovery, or stability, because the follow-up was incomplete and there was no untreated growth control across the broad 5–17-year age range. In the surgically assisted cohort, SNA–Me and SOR–Me changed by −1.66 and −1.23 mm at T1–T2, respectively, but these exploratory observations (*n* = 18) do not support conclusions about persistent vertical effects across skeletal patterns. The different populations and protocols in conventional RPE [11], surgically assisted expansion [23], and transverse–sagittal approaches [24] also limit direct comparison.

Dental-cast measurements complement, rather than duplicate, cephalometric outcomes. Both cohorts showed substantial T0–T1 maxillary intermolar widening on casts (+6.83 mm orthopedic; +5.71 mm surgical), whereas the T1–T2 upper-intermolar cast changes were small (+0.44 and −0.41 mm, respectively). Corresponding cephalometric changes were +0.42 and −0.38 mm. The numerical proximity of these aggregates mean changes must not be interpreted as demonstrated intermethod agreement: the anatomical landmarks differ, and although the surgical T2 cast and cephalometric subsets comprise the same 18 patients, the orthopedic cast subset contains only 18 of 20 patients with T2 cephalograms. Agreement requires a patient-level paired-method comparison, not a comparison of group means. The small negative T1–T2 changes observed in the surgically assisted cohort (−0.38 mm on cephalometric measurements and −0.41 mm on dental casts) may reflect minor post-expansion dentoalveolar adaptation, occlusal settling, or a limited degree of transverse relapse, consistent with previously reported post-SARPE changes [33,34]. However, these potential mechanisms were not directly investigated, and the small magnitude of the observed changes, together with incomplete longitudinal follow-up, does not permit definitive conclusions regarding clinically relevant relapse or long-term treatment stability [33,34].

Differences in growth status, clinical indication, and retention duration (6 months after orthopedic RPE versus 8 months after surgically assisted expansion) limit cross-cohort interpretation, especially because T1 was obtained immediately after appliance removal. Accordingly, this study reports parallel within-cohort observations, rather than a test of comparative treatment effectiveness. Published syntheses, similarly, describe variable skeletal and dentoalveolar contributions and heterogeneous long-term evidence [35,36].

The reported 95% confidence intervals quantify precision of the available within-cohort mean change scores; they do not demonstrate agreement between measurement methods or equivalence of treatments. At T2, cephalometry was available for 20/54 orthopedic and 18/27 surgical patients, while dental casts were available for 18 patients in each cohort. The 18 surgical records provided both modalities, whereas 18 orthopedic cast records formed a subset of the 20 cephalometric follow-up records. This overlap makes patient-level paired evaluation conceptually feasible where the original data are available, but it has not been performed in the present summary analyses. Missing T2 records may introduce attrition bias, and exploratory changes do not establish long-term stability or between-treatment differences.

From a clinical perspective, both skeletal and dental transverse outcomes should be considered when assessing expansion, while acknowledging that they represent distinct anatomical measurements. In the orthopedic cohort, the +1.57 mm mandibular intercanine increase during T0–T1, despite the appliance being confined to the maxillary arch, may partly reflect normal dental eruption, mixed-dentition development, altered interarch contact relationships, or spontaneous dentoalveolar adaptation following expansion. Likewise, the additional +1.38 mm maxillary intercanine change among children with T2 casts could be influenced by the eruption and alignment of permanent canines. However, eruption stages, tooth-specific tipping, and age-stratified changes were not assessed, and, in the absence of a growth control, these explanations remain hypothetical, not verified causal mechanisms. In the adult surgical cohort, the larger dental-cast widening than the skeletal MXR–MXL change at T0–T1 may represent combined skeletal and dentoalveolar contributions, including possible anchorage-tooth inclination; tipping was not measured directly, and different landmarks prevent quantification of those components from the present results [4,10,31,32].

Future craniofacial-regeneration research may investigate whether microenvironment-responsive biomaterials can complement mechanically induced bone remodeling [38].

Strengths include cohort-specific protocols, common operator supervision, skeletal maturity assessment, multiplanar cephalometry complemented by dental casts, quantitative measurement reliability, and a precision-based a priori sample-size calculation for the primary T0–T1 summaries.

Important limitations govern interpretation. The retrospective, nonrandomized design permits selection and information bias and residual confounding; treatment groups differ by age, skeletal maturity, clinical indication, and retention duration, precluding causal between-cohort comparisons. In the orthopedic cohort (ages 5–17 years), normal growth and dental eruption may explain part of the measured change, and no untreated growth control or stage-specific analyses were available. T2 records were incomplete (cephalometry: 20/54 orthopedic and 18/27 surgical patients; casts: 18 patients per cohort) and were not included in the sample-size planning; analyses used available cases without imputation. Selection into the follow-up subset and attrition bias may therefore influence even seemingly precise estimates. The two methods used different anatomical landmarks, with incomplete modality overlap in the orthopedic cohort, so method agreement was not formally assessed. Two-dimensional cephalometry is sensitive to projection, magnification, landmark superimposition, and head positioning, and some center-related measurement or follow-up variation cannot be excluded. Patient-level cumulative expansion amounts and dental eruption stages were incompletely available; potential tipping was not assessed. Accordingly, the T1–T2 findings are exploratory, and cannot be treated as definitive evidence of long-term stability or relapse.

## 5. Conclusions

Within each of two clinically distinct cohorts, orthopedic rapid palatal expansion in growing patients and surgically assisted expansion in skeletally mature adults were accompanied by T0–T1 maxillary transverse widening. The descriptive follow-up data show small T1–T2 maxillary intermolar changes among available cases: +0.42 mm (cephalometry) and +0.44 mm (casts) in the orthopedic cohort, and −0.38 mm and −0.41 mm, respectively, in the surgically assisted cohort. These aggregate changes cannot demonstrate agreement between methods or establish the course of changes in patients without follow-up.

The retrospective, nonrandomized design, differences in skeletal maturity and treatment indications, incomplete T2 observations, and measurement limitations preclude causal or comparative-effectiveness inferences and definitive conclusions on long-term stability or relapse. The observed mandibular and maxillary intercanine changes in growing patients may reflect normal dentition development, as well as treatment-associated adaptation; these mechanisms were not tested.

Prospective controlled studies with prespecified follow-up windows, complete patient-level paired radiographic and dental-cast records, and adequate longitudinal samples are needed to confirm these observations and explain the separate skeletal and dentoalveolar contributions.

## Figures and Tables

**Table 1:** Baseline clinical characteristics and follow-up availability of the two cohorts.

Characteristic	Surgically Assisted Cohort	Orthopedic Cohort
Patients, *n*	27	54
Age range, years	21–35	5–17
Sex, female/male	15/12	27/27
Treatment/inclusion period	January 2024–December 2025	January 2024–December 2025
Skeletal maturity classification	Nongrowing/skeletally mature (CVM; hand–wrist assessment when clinically indicated)	Growing/remaining growth potential (CVM distribution: approximately 30% CS2, 50% CS3, 20% CS4; hand–wrist assessment when clinically indicated)
Skeletal Class I/II/III	6/3/9 among 18 classified patients	21/15/18
Expansion protocol	Surgically assisted rapid maxillary expansion	Orthopedic rapid palatal expansion
In situ retention with expander	8 months	6 months
T0–T1 records included	27	54
Vertical pattern, deep bite/normodivergent/open bite	3/6/9 among 18 classified patients	21/9/24
Baseline transverse diagnosis	Transverse maxillary deficiency with posterior crossbite (all patients)	Transverse maxillary deficiency with posterior crossbite (all patients)
Crossbite laterality/severity	Not systematically coded in the analytical dataset	Not systematically coded in the analytical dataset
Patient-level cumulative expansion amount	Individualized; final cumulative activation not retained in the analytical dataset	Individualized; final cumulative activation not retained in the analytical dataset
T2 cephalometric records available, *n*	18	20
T2 dental-cast records available, *n*	18	18
T2 assessment interval	12 months after appliance removal (T1)	12 months after appliance removal (T1)

## Data Availability

The data presented in this study are available on reasonable request from the corresponding author.

## Supplementary Materials

The following supporting information can be downloaded at: https://www.mdpi.com/article/10.3390/bioengineering13101221/s1, Table S1: Intra- and interoperator reliability of cephalometric and dental-cast measurements.
